# CONTRAST SENSITIVITY AND BRAIN NETWORK COMMUNITY STRUCTURE IN THE BRAIN NETWORKS AND MOBILITY STUDY

**DOI:** 10.1093/geroni/igad104.2102

**Published:** 2023-12-21

**Authors:** Atalie Thompson, Alexis Tanase, Michael Miller, Jeff Williamson, Christina Hugenschmidt, Haiying Chen, Stephen Kritchevsky, Paul Laurienti

**Affiliations:** Wake Forest University School of Medicine, Winston-Salem, North Carolina, United States; Wake Forest University School of Medicine, Winston-Salem, North Carolina, United States; Wake Forest University School of Medicine, Winston Salem, North Carolina, United States; Wake Forest School of Medicine, Winston Salem, North Carolina, United States; Wake Forest University School of Medicine, Winston-Salem, North Carolina, United States; Wake Forest University School of Medicine, Winston-Salem, North Carolina, United States; Wake Forest School of Medicine, Winston Salem, North Carolina, United States; Wake Forest University School of Medicine, Winston-Salem, North Carolina, United States

## Abstract

Contrast sensitivity (CS) is the ability to perceive differences in shades of light and dark, which is important for pattern recognition and depth perception. We have previously shown that impaired CS is associated with poor performance on the expanded short physical performance battery (eSPPB), and that performance on the eSPPB is associated with sensory motor cortex (SMC) connectivity at rest. We hypothesized that brain networks are important to the relationship of CS with eSPPB. Participants (n=192) were cognitively unimpaired older adults (mean age=76.5±4.7 years; 56.5% female, 9.4% black) with good visual acuity who completed CS testing. We applied graph theory to characterize the cross-sectional relationship of binocular CS to functional brain networks generated from fMRI both at rest and during a motor imagery (MI) task and determined the spatial patterns of SMC, visual (VIS) and default mode (DMN) network connectivity. Regression analyses were used to assess associations of CS with brain network connectivity without and with adjustment for sex and eSPPB. Lower CS (p=0.009) was significantly associated with degraded SMC connectivity at rest even in models adjusted for eSPPB and sex. Similarly, lower CS was significantly associated with degraded VIS and DMN connectivity during the MI task (all p< 0.005) when controlling for eSPPB and sex. These results indicate that low CS and poor eSPPB are both associated with connectivity in the SMC at rest and with VIS and DMN during MI in older adults. These findings may be important for understanding the relationship between age-related visual and mobility dysfunction.

